# A Multidisciplinary Investigation of the First Chikungunya Virus Outbreak in Matadi in the Democratic Republic of the Congo

**DOI:** 10.3390/v13101988

**Published:** 2021-10-03

**Authors:** Anja De Weggheleire, Antoine Nkuba-Ndaye, Placide Mbala-Kingebeni, Joachim Mariën, Esaie Kindombe-Luzolo, Gillon Ilombe, Donatien Mangala-Sonzi, Guillaume Binene-Mbuka, Birgit De Smet, Florian Vogt, Philippe Selhorst, Mathy Matungala-Pafubel, Frida Nkawa, Fabien Vulu, Mathias Mossoko, Elisabeth Pukuta-Simbu, Eddy Kinganda-Lusamaki, Wim Van Bortel, Francis Wat’senga-Tezzo, Sheila Makiala-Mandanda, Steve Ahuka-Mundeke

**Affiliations:** 1 Outbreak Research Team, Institute of Tropical Medicine, 2000 Antwerp, Belgium; Joachim.Marien@uantwerpen.be (J.M.); bdesmet@itg.be (B.D.S.); florianvogt@hotmail.com (F.V.); pselhorst@itg.be (P.S.); wvanbortel@itg.be (W.V.B.); 2 Department of Virology, National Institute of Biomedical Research, B.P. 1197 Kinshasa I, Democratic Republic of the Congo; antoinnkuba@gmail.com (A.N.-N.); mbalaplacide@gmail.com (P.M.-K.); kibain3@gmail.com (E.K.-L.); fridankawa@gmail.com (F.N.); elisepukuta@gmail.com (E.P.-S.); eddylusamaki@gmail.com (E.K.-L.); shemakiala@yahoo.fr (S.M.-M.); amstev04@yahoo.fr (S.A.-M.); 3 Department of Medical Biology, University of Kinshasa, B.P. 127 Kinshasa IX, Democratic Republic of the Congo; donatienmangala@gmail.com (D.M.-S.); mathymatungala01@gmail.com (M.M.-P.); cedricvulu2014@gmail.com (F.V.); 4 TransVIHMI, Institut de Recherche pour le Développement, Institut National de la Santé et de la Recherche Médicale (INSERM), Montpellier University, 34090 Montpellier, France; 5 Department of Entomology, National Institute of Biomedical Research, B.P. 1197 Kinshasa I, Democratic Republic of the Congo; gillonilombe@yahoo.fr (G.I.); bimbunt@gmail.com (G.B.-M.); tezzowatsenga@gmail.com (F.W.-T.); 6 Global Health Institute, Antwerp University, 2000 Antwerp, Belgium; 7 The Kirby Institute, University of New South Wales, Sydney, NSW 2052, Australia; 8 National Centre for Epidemiology and Population Health, Research School of Population Health, College of Health and Medicine, Australian National University, Canberra, ACT 2601, Australia; 9 Direction de Lutte contre la Maladie, Ministry of Health, B.P. 3040 Kinshasa I, Democratic Republic of the Congo; mossokomathias@gmail.com

**Keywords:** chikungunya, outbreak investigation, multidisciplinary, Democratic Republic of the Congo, *Aedes albopictus*

## Abstract

Early March 2019, health authorities of Matadi in the Democratic Republic of the Congo alerted a sudden increase in acute fever/arthralgia cases, prompting an outbreak investigation. We collected surveillance data, clinical data, and laboratory specimens from clinical suspects (for CHIKV-PCR/ELISA, malaria RDT), semi-structured interviews with patients/caregivers about perceptions and health seeking behavior, and mosquito sampling (adult/larvae) for CHIKV-PCR and estimation of infestation levels. The investigations confirmed a large CHIKV outbreak that lasted February–June 2019. The total caseload remained unknown due to a lack of systematic surveillance, but one of the two health zones of Matadi notified 2686 suspects. Of the clinical suspects we investigated (*n* = 220), 83.2% were CHIKV-PCR or IgM positive (acute infection). One patient had an isolated IgG-positive result (while PCR/IgM negative), suggestive of past infection. In total, 15% had acute CHIKV and malaria. Most adult mosquitoes and larvae (>95%) were *Aedes albopictus*. High infestation levels were noted. CHIKV was detected in 6/11 adult mosquito pools, and in 2/15 of the larvae pools. This latter and the fact that 2/6 of the CHIKV-positive adult pools contained only males suggests transovarial transmission. Interviews revealed that healthcare seeking shifted quickly toward the informal sector and self-medication. Caregivers reported difficulties to differentiate CHIKV, malaria, and other infectious diseases resulting in polypharmacy and high out-of-pocket expenditure. We confirmed a first major CHIKV outbreak in Matadi, with main vector *Aedes albopictus*. The health sector was ill-prepared for the information, surveillance, and treatment needs for such an explosive outbreak in a CHIKV-naïve population. Better surveillance systems (national level/sentinel sites) and point-of-care diagnostics for arboviruses are needed.

## 1. Introduction

Chikungunya virus (CHIKV) is a single-stranded RNA virus of the genus *Alphavirus*, in the Togaviridae family, originating from sub-Saharan Africa, where sylvatic and urban cycles are recognized. The sylvatic transmission cycle involves a number of forest-dwelling mosquitoes such as *Aedes africanus, Aedes luteocephalus,* and *Aedes furcifer-taylori,* and wild primates wherefrom spill-over to humans can occur. The urban cycle involves the vectors *Aedes aegypti* originating from the African continent and *Aedes albopictus* imported from Southeast Asia, which was only detected for the first time in the Democratic Republic of the Congo (DRC) in 2016 [1,2]. The virus is transmitted to humans by day-time bites of these mosquitoes, which may also transmit dengue and other arboviruses of public health importance.

CHIKV infection has an incubation period of 2 to 10 days with an average of 3 days, and typically causes abrupt onset of high fever and polyarthralgia, also referred to as chikungunya fever. Other symptoms include headache, myalgia, arthritis, conjunctival hyperemia, nausea/vomiting, and maculopapular rash [3,4]. While most patients recover fully and overall lethality is low (1–2% of cases), joint pain may persist for months or even years [5]. CHIKV infection causes antibody responses that confer long-term, possibly lifelong protection [6,7]. Among populations with no prior immunity, CHIKV outbreaks can be explosive, and attack rates between 10 and 70% have been documented [8,9,10]. Because of the wide range of clinical symptoms, chikungunya fever is often mistaken for other tropical diseases such as malaria or dengue. The virus can be detected by PCR in the first week after fever onset. Antibodies, mostly measured by lab-based enzyme-linked immunosorbent assays (ELISA), are detectable from as early as three days after symptom onset [11,12]. Both tests are expensive and require trained personnel, which is often limited in areas prone to CHIKV outbreaks.

**Figure 1:**
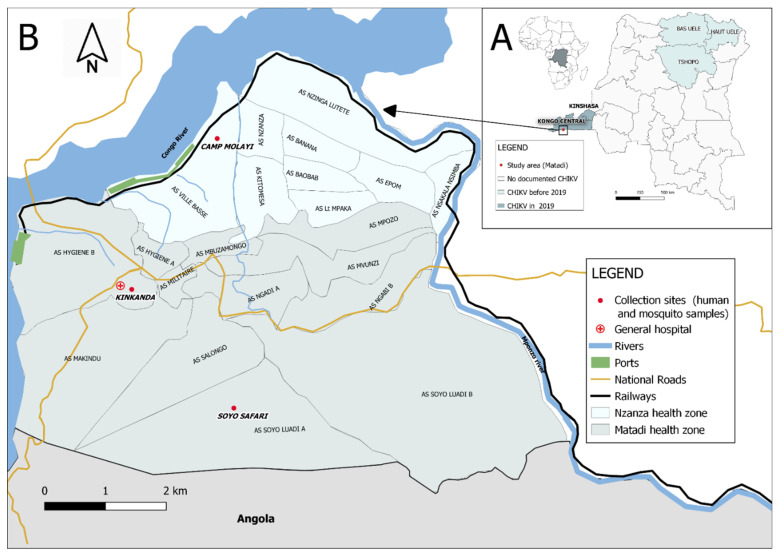
(**A**) Map of the Democratic Republic of the Congo (DRC) indicating the provinces where chikungunya virus outbreaks have occurred up to 2019 (including the Matadi study area indicated with the red dot). (**B**) Map of Matadi (study area) in Kongo Central province in DRC. The red dots indicate the places where human and mosquito samples were collected.

In the DRC, CHIKV was first identified in the north-eastern part of the country, in 1958, in Doruma village (Haut-Uéle Province), and in 1960, in Bondo territory (Bas-Uélé Province) [13,14]. Since then, no further circulation had been documented until 1998, when 12 patients tested positive for CHIKV IgM antibodies in Kisangani, Tshopo province (Figure 1A) [15]. Subsequently, consecutive CHIKV outbreaks were reported in the capital Kinshasa, with an estimated 50,000 infected cases in the period of 1999–2000. The main affected areas were the urban communes of Matete and Limete (mostly in quartier Kingabwa) and the eastern rural N’sele commune (quartier Kinkole) [16]. The western part of Kinshasa (Mont Ngafula and Mbinza Meteo health zones) experienced its first documented outbreak in 2012 [17].

In November 2018, alerts arose again from this area, and in mid-February 2019, CHIKV infections were laboratory-confirmed, but no official outbreak declaration was reported by the Ministry of Health (MoH) [18,19]. Due to the lack of official declaration, and as arboviruses were not part of the mandatory notifiable diseases in the MoH surveillance system in DRC, a coherent epidemiological follow-up of the spread of the outbreak was challenging. Alerts were also gradually reported from the neighboring health zones in Kongo Central province. In this province, CHIKV had never been identified before [20]. Early March 2019, health authorities of Matadi city (located about 330 km from Kinshasa in the western end of Kongo Central province) notified a sudden increase in patients with acute fever and arthralgia. Based on the clinical data, they suspected an expansion of the CHIKV outbreak and requested the ‘Institut National de Recherche Biomédicale’ (INRB) for an evaluation. In response, a multidisciplinary outbreak investigation team, composed of members from INRB and the Institute of Tropical Medicine-Antwerp (ITM) and mandated by the Ministry of Health (MoH) of DRC, was dispatched to Matadi, on 17 March 2019. The aim was to investigate the situation in order to confirm CHIKV as the causal pathogen, to assess the epidemiological, clinical, laboratory, and entomological characteristics of the outbreak and to gain knowledge on the perception of the population regarding the outbreak. In this paper, we report on the findings of this outbreak investigation and review the lessons learned.

## 2. Materials and Methods

### 2.1. Outbreak Investigation Setting and Teams

The outbreak investigation was performed between 17 and 23 March 2019 (epi week 12) in Matadi by a multidisciplinary team, including clinical epidemiologists, microbiologists, entomologists, and a social scientist, together with local MoH teams. Matadi is a densely populated portal city located in the Kongo Central province near the DRC-Angola border at a 5.5 h drive from Kinshasa. The city consists of three communes (Matadi 60 km^2^, Nzanza 51 km^2^, and Mvuzi 24 km^2^) and has approximately 461.537 inhabitants (source DHIS2). It has a tropical climate with a long rainy season from November to May. At sanitary level, the city is covered by two health zones (HZ), the Matadi HZ (subdivided in 12 ‘aires de santé’, 277.541 inhabitants) at the western/central part of town and the Nzanza HZ (9 ‘aires de santé’, 168.993 inhabitants) on the south-eastern side (Figure 1B). Kongo Central province is, as most of DRC, located in an area with high levels of malaria transmission [21]. When the team arrived in Matadi, none of the clinical CHIKV suspects had yet been confirmed by diagnostic testing.

### 2.2. Data Collection

#### 2.2.1. Epidemiological Investigations

The provincial and health zone MoH authorities were consulted upon arrival in Matadi. They provided data on the initial alerts, the spread, and the number of weekly clinical suspects notified by health zone. Notification of clinical suspects was encouraged on the initiative of the provincial MoH from the last week of February 2019 (epi week 9) but remained incomplete as CHIKV was not part of the routine surveillance system. They recommended the following clinical case definition: a patient presenting with sudden onset of high fever and severe arthralgia, with or without a transient skin rash, is to be considered as a clinical acute CHIKV suspect. The MoH surveillance teams of Matadi HZ continued to share the available weekly CHIKV clinical suspect notification data to the outbreak investigation team, up to the end of June 2019.

#### 2.2.2. Clinical and Laboratory Investigations

Based on the MoH briefing, the investigation team identified two high-incidence areas (Soyo Safari, in aire de santé Makindu of Matadi HZ, and the Police Camp Molayi, in aire de santé Ville Basse, Nzanza HZ—Figure 1B), where mobile clinics were organized targeting persons with recent (since maximum five days) onset of fever and/or arthralgia. These mobile clinics operated in the immediate neighborhood of the main primary healthcare center serving the area with support and presence of the health staff of those centers. Communities were informed by community healthcare workers about the purpose and practical details of these mobile clinics. Patients were offered a clinical consultation, malaria testing by antigen rapid diagnostic test (CareStart™ Malaria Pf (HRP2) or SD Bioline™ Malaria Pf/Pan (HRP2/pLDH) as supplied by the national malaria program) on a capillary finger prick sample, a blood draw (5 mL EDTA tube) for confirmation of CHIKV infection, and treatment (symptomatic treatment and, if malaria RDT positive, antimalarials).

In parallel, the medical teams of the Provincial Reference Hospital and the nearby health center CEZO (both located in the quartier Ville Haute in the Matadi HZ, aire de santé Hygiène B) were sensitized to collect similar data (clinical data, malaria diagnosis, and venous blood sample) of clinical suspects presenting at their outpatient services, during the stay of the outbreak investigation team in Matadi.

Individual patient data were collected using a standardized outbreak investigation form for suspect CHIKV cases. These forms contained socio-demographic variables, clinical signs and symptoms, and malaria testing results.

The blood samples were immediately stored at 4 °C on site (during maximum four hours), and further kept in a −20 °C freezer until diagnostic testing for CHIKV infection, which was performed at the Virology Department of INRB in Kinshasa. Both CHIKV RT-PCR testing and serology (IgM and IgG) were performed in parallel on the samples of the clinical suspects attended at the mobile clinic, and sequentially (RT-PCR followed by serology if RT-PCR negative) for the samples of the patients seen in the outpatient department.

RNA extraction for PCR analysis was carried out manually with the QIAamp Viral RNA Mini Kit (QIAGEN) on 140 µL plasma collected in EDTA whole blood tubes after passive sedimentation by gravity. RNA from phocine distemper virus (PDV) was added to all samples as an internal RNA extraction control and PCR inhibition control. A CHIKV-specific RT-qPCR was then performed with 5 µL RNA in a 25 µL reaction using the iTaq universal probes one-step kit from Bio-Rad by amplifying a 77 bp part of the non-structural protein 1 (NSP-1) gene with primers and probes adapted from Panning et al. 2008 [22,23]. Cycling conditions were 10 min at 50 °C, a denaturation step of 5 min at 95 °C, followed by 50 cycles of 10 s at 95 °C and 30 s at 60 °C. A PDV RT-qPCR was run in parallel [24]. In every RNA extraction batch, a negative control was included, and in every PCR run, a negative control was used.

The IgM and IgG antibodies for CHIKV were detected using the enzyme-linked immunosorbent assay (ELISA) IgM and IgG test from Euroimmun, Lübeck, Germany. As per manufacturer instructions, an optical density (OD) ratio on ELISA IgM or ELISA IgG > 1 was considered as a positive ELISA IgM or IgG result. A positive CHIKV PCR and/or positive CHIKV IgM result defined an acute CHIKV infection for the clinical suspects sampled during this investigation. A positive CHIKV IgG result in the absence of CHIKV RNA and/or IgM antibodies was considered a past CHIKV infection.

#### 2.2.3. Entomological Investigations

Both adult and immature stages of Aedes mosquitoes were sampled in three neighborhoods with suspected CHIKV cases (Soyo Safari, Kinkanda, and Camp Molayi) (see Figure 1B). Adult mosquitoes were trapped using six battery-powered BG-sentinel traps and two Prokopack aspirators. The traps were placed in vegetation surrounding houses (preferably in the shade), activated in the morning and emptied in the evening. Aspirator collections were made from vegetations only and were performed in the late afternoon. Water-holding containers in the neighborhoods were inspected for larvae (both indoors and outdoors), reported on entomological survey forms, and sampled if positive. Collections were made during one day for each site. Larvae were reared to adults in the laboratory for morphological identification following Walter Reed’s identification keys: we looked at the characteristic patterns on the scutum (white longitudinal stripe for *Ae. albopictus* and lyre-shaped white markings for *Ae. aegypti*) and the white/black patterns of the tarsi on the legs [25]. To ensure the correct identification of *Aedes albopictus* in the area (as this species was never detected before in Matadi), we selected two individual mosquitoes for DNA barcoding (a technique based on the amplification of the partial mitochondrial cytochrome c oxidase subunit I (COI) gene), which we excluded from the pools and preserved in separate tubes [26]. Sanger sequencing of the 658-base pair barcode and phylogenetic analyses of these specimens followed laboratory protocols and methodologies described in [26] and by Mariën et al. (amplicons obtained from the Matadi specimens were included in the phylogenetic tree of the latter reference) [27]. In short, PCR amplicons (EPPO 2016, LCO1490, and HCO2198 universal primers) and negative controls were checked on an agarose gel, sequenced in both directions, and compared against BOLD Identification System with Species Level Barcode Records.

Larvae indices were calculated to estimate the level of infestation in the affected areas [28]:

- Container index: number of containers positive for immature stages of *Aedes* spp. per 100 inspected containers.
- House index: number of houses positive for at least one container with immature stages of *Aedes* spp. per 100 inspected houses.
- Breteau index: number of containers positive for immature stages of *Aedes* spp. per 100 inspected houses.

All adult mosquitoes were killed by ethanol inhalation, identified, and separated by sex. The mosquitoes were pooled according to sampling site, sex, stage during capture (adult/larvae), and species in Eppendorf tubes with RNA shield for viral preservation (max. 50 individuals per tube). The mosquito pools were immediately homogenized in the field using Zymo Research bashing beads (vortex for 2 min). They were stored at 4 °C for one week during the field investigation and further kept at −20 °C at INRB in Kinshasa. All pooled samples were screened for the presence of CHIKV using the Zymo quick DNA/RNA pathogen extraction kit and RT-qPCR. All RT-qPCR reactions were performed in duplo and samples were considered to be positive if both reactions gave the same Ct-value (±1), as described by Selhorst et al. [19].

#### 2.2.4. Qualitative Data about Perceptions and Behaviors Related to the Outbreak

To understand the community perceptions regarding the cause of the outbreak and to explore community behaviors associated with the illness, the team conducted semi-structured interviews with a convenient sample among adult patients consulting at the mobile clinic sites in Soyo Safari (*n* = 20) and Camp Molayi (*n* = 28) (see Appendix A: interview guide). Further, provincial and local health authorities, as well as doctors and nurses (*n* = 12) from the above-listed health facilities, were interviewed focusing on medical care, prevention measures, access, and context.

### 2.3. Data Analysis

The epidemiological data of Matadi HZ (weekly notified suspected cases per ‘aire de santé’) were used to construct an epidemic curve displaying the duration and extent of the outbreak.

Based on the clinical data and samples collected during the field investigations, proportions of patients with acute CHIKV infection, coinfection with malaria, and past CHIKV exposure were calculated. Socio-demographic characteristics and clinical features were summarized using medians and interquartile ranges for continuous variables and percentages for categorical variables. Subgroups (confirmed acute CHIKV infection vs. those without acute CHIKV infection) were compared by Fisher’s exact tests for categorical variables and Wilcoxon rank sum tests for continuous variables. An alpha error of 5% was considered statistically significant. Analyses were conducted with Stata IC 15.0 (StataCorp, College Station, TX, USA).

CHIKV infection rates in pooled samples of collected mosquitoes were estimated based on a maximum likelihood (ML) estimation implemented in the Microsoft^®^ Office Excel© Add-In package from the Centers for Disease Control and Prevention, U.S.A [29].

Qualitative data were analyzed by thematic content analysis. Common themes were manually determined via an inductive approach.

### 2.4. Ethical Considerations

This outbreak investigation including sample collection was exempted from review by the Ethics Committee of the School of Public Health of the University of Kinshasa (DRC) since it was an emergency response investigation led by the DRC national reference laboratory for outbreak investigation, the ‘Institut National de Recherche Biomédicale’. The investigation was approved by and conducted in collaboration with the Ministry of Health. The Ethics Committee of the School of Public Health of the University of Kinshasa (DRC, ESP/CE/138/2020) approved that the de-identified data of the outbreak investigation could be used for scientific publication.

## 3. Results

### 3.1. Epidemiological Description of the Outbreak

**Figure 2:**
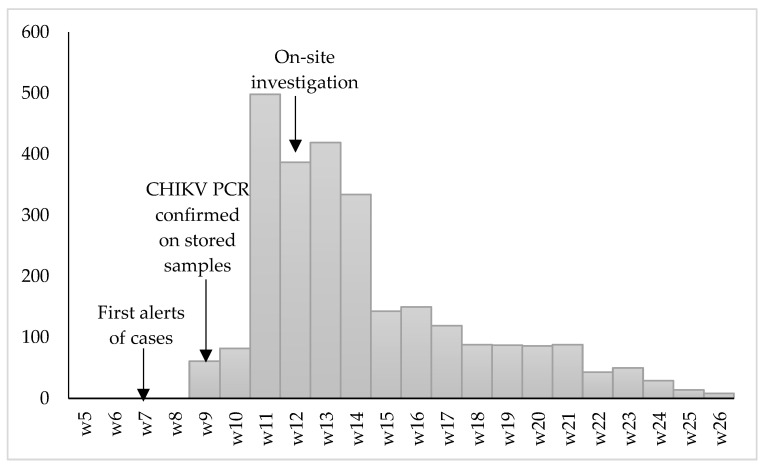
Epidemic curve based on weekly notification data of chikungunya fever suspected cases in Matadi health zone, by 2019 epi-weeks. Note: Chikungunya virus infections are not part of the diseases/syndromes of the national standard surveillance system in DRC. Notification was encouraged by the health authorities, but not obligatory, and was expected therefore to be incomplete.

In Matadi HZ, 2686 clinical suspected cases were notified to the MoH between the last week of February 2019 (i.e., after the provincial MoH spread a notice with a clinical case definition to encourage health structures to start notifying) and end of June 2019 (epi week 26) (see Figure 2). The outbreak started mid-February 2019 (epi week 6) by alerts from health structures in Makindu aire de santé in Matadi HZ and gradually decreased in intensity toward the dry season in May–June 2019. The Makindu, Hygiène A/B, Soyo Luadi A/B, Militaire, and Mpozo aire de santé of Matadi HZ notified most cases. Similar data were not available for Nzanza HZ.

### 3.2. Clinical Characteristics and Laboratory Results of the Chikungunya Fever Suspected Cases

During the investigation, clinical data were collected for 220 clinical suspects. Of these, 162 patients were seen during the active screening via mobile clinics, and 58 in the outpatient department of the Provincial Reference Hospital and nearby health center. The majority was female (61.8%), and the median age was 23 years (IQR 10-38) (Table 1). The median duration of symptoms was three days (IQR 2-5). In total, 183 (83.2%) patients had a confirmed acute CHIKV infection based on the RT-PCR or IgM serology; 44 patients (20%) had a positive malaria RDT result; and 15 percent of the clinical suspects had an acute CHIKV/malaria co-infection. Malaria positivity was not significantly different between patients with or without confirmed acute CHIKV (*p* = 0.26). However, overall, malaria positivity was significantly higher among those below 15 years of age (31.2%, *p* = 0.008) as compared with older patients (14.7%). The age group of 10–15 years had the highest coinfection rate (31.3%); 27 patients (10.4% of those aged below 15 years and 14.0% of those equal or above 15 years) had an acute fever and/or arthralgia syndrome, which remained unexplained after negative CHIKV and malaria testing.

**Figure 3:**
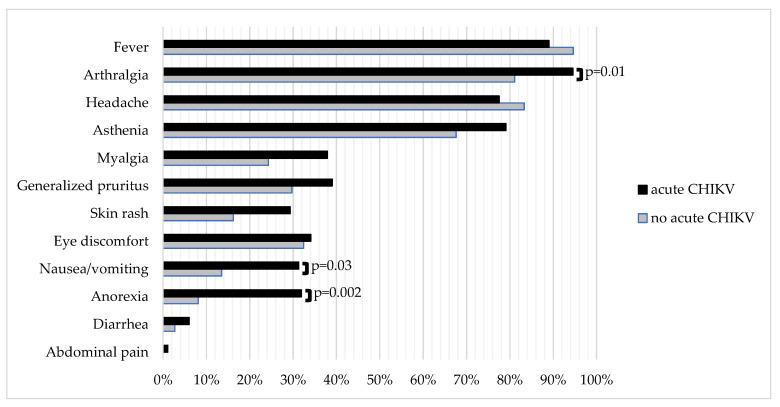
Comparison of frequency of clinical signs/symptoms between patients with acute CHIKV (N = 182) and without acute CHIKV (N = 37) infection. Note: Symptom data were missing for one patient with acute CHIKV infection. *p*-values if below 0.05.

Symptoms such as arthralgia, anorexia, and nausea/vomiting were significantly more frequent among those with an acute CHIKV infection compared with those without. Joint pain of acute CHIKV patients was most often located at the knees (60.4%, 102/169), ankles (47.3%, 80/169), wrists (34.9%, 59/169), and/or elbows (24.3%, 41/169); 99 patients presenting with arthralgia had complaints in three or more different joint locations (11/30 patients without acute CHIKV infection and 88/169 patients with acute CHIKV, *p* = 0.16). Detailed data on location of arthralgia were missing for three patients. Further details on the frequency of symptoms in both groups are shown in Figure 3.

**Figure 4:**
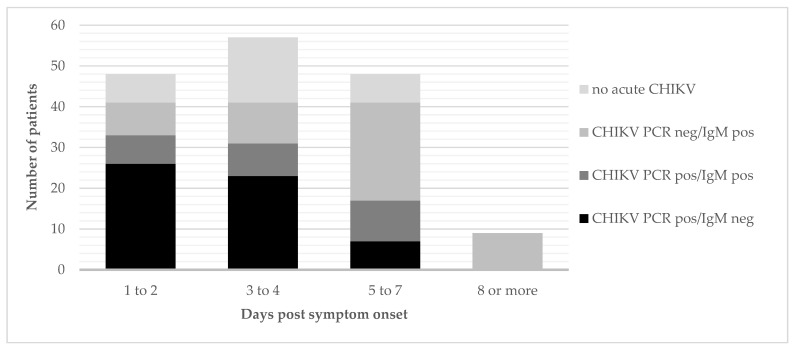
Classification by acute CHIKV status and diagnostic results of the clinical suspects attending the mobile clinics (N = 162), by days post symptom onset.

The samples of the clinical suspects attended by the mobile clinics were tested by RT-PCR and ELISA in parallel. Of the 132 mobile clinic attendants diagnosed with acute CHIKV infection, 56 tested positive by RT-PCR only, 25 tested positive by both RT-PCR and serology (IgM), and 51 were positive for IgM only. The yield of additional acute CHIKV diagnoses by IgM testing increased with days post-symptom onset—less or equal to two days: 20%; three to four days: 24%; five to seven days: 59%; and 100% from 8 days onward (Figure 4). Of the RT-PCR-positive samples, the Ct value increased with days post-symptom onset (Spearman r = 0.39, *p* < 0.001)—less or equal to two days: median Ct = 18.7; three to four days: median Ct = 22.0; and five or more days: median Ct = 26.9. Only one person (male adult from Camp Molayi) had an isolated CHIKV IgG-positive result indicative of past exposure.

### 3.3. Entomological Study

All adult captures and more than 95% of larvae were identified as *Ae. albopictus*, of which high levels of infestation (House, Breteau, and Container indexes) were noted (Table 2). Given that the number of *Ae. aegypti* was low, only the *Ae. albopictus* pools were checked for the presence of CHIKV. We detected the virus in 6/11 of the adult pools, and in 2/15 of the larvae (reared to adult) pools (Table 3). DNA barcoding confirmed the identification as *Ae. albopictus* (100% sequence similarity with the reference database) for the two specimens that were checked molecularly (sequences deposited in Genbank with accession numbers: MZ673322-MZ673323). An interesting observation was that 2/6 CHIKV-positive adult pools contained only male mosquitoes, and two larval pools were CHIKV-positive (albeit with high Ct-values). These individuals could not have taken a blood meal, suggesting that they have become infected transovarially.

### 3.4. Perceptions and Behaviors Related to the Outbreak

Patient and caregiver interviews revealed that initially patients sought care in the formal health structures, but that they gradually shifted to self-diagnosis and self-medication based on the advice/prescriptions of neighbors or other family members who presented similar symptoms. Community members expressed dissatisfaction with the fact that consultations and treatment were not offered for free as for malaria (“*Malaria is also a disease transmitted by mosquitoes, and for that care and treatment is free of charge…so if chikungunya is also transmitted by mosquitoes …treatment should also be for free, no?*”) or during other outbreaks.

As to the origin or cause of the outbreak, several conspiracy theories were circulating fed by a general disbelief that the mosquitoes, which were there with them in the community for a long time, all of a sudden would be causing disease and even an outbreak. Some were thinking that “*satanic mosquitoes had infiltrated from a neighboring country*”; others rather found ground in existing national/regional political tensions (“*It must be politicians who are behind this in an attempt to destroy the Kongo people*”). In the police camp (Camp Molayi), a rumor circulated that “*Tanzanian researchers had created and sent the chikungunya mosquitoes to them as an experiment*”, but others there also made the link with the general bad sanitation situation in the camp (very crowded, leaking water pipes and stagnating water, and lack of toilets).

Driven by the belief that these could not be the normal mosquitoes that were causing this, the community turned mostly to prayers and use of traditional bracelets as means of prevention. The bracelets were made of thin twigs mostly from the plant/tree, which is also used for natural toothbrushes, and were believed to protect against the ‘bewitched day-time’ mosquitoes. They were sold at a price ranging 0.2 to 1 dollar depending on the socio-economic status of the neighborhood [30]. A same kind of bracelet had also been used during the 2016 yellow fever outbreak. However, already in mid-March 2019, trust in this method started to decrease as people wearing the bracelets also fell sick.

**Figure 5:**
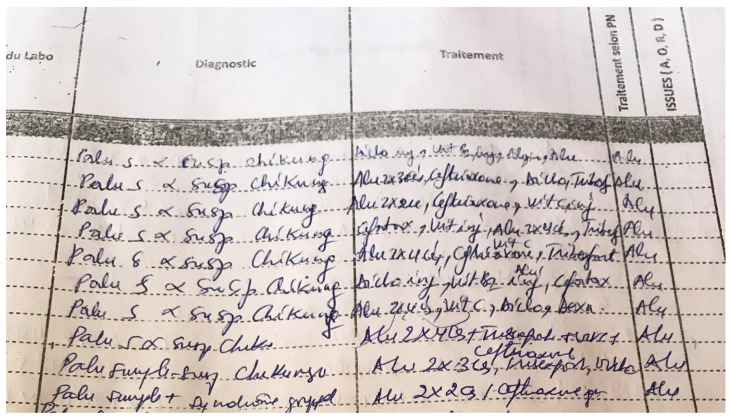
Snapshot—part of consultations of 28 February 2019—from a consultation register in the Matadi health zone.

Health workers highlighted the challenge to differentiate between malaria episodes, acute CHIKV infections (or both) and other etiologies, when based only on clinical features, and a malaria RDT as diagnostic tools. Negative malaria RDT results were not always considered conclusive/trustworthy, especially in patients with very high fever. Caregivers referred to the possibility of false negative RDTs in case of severe malaria, and not wanting to risk missing such a malaria episode. A snapshot of a page of the outpatient register from a health center in Matadi HZ demonstrates this difficulty (Figure 5). Polypharmacy (antimalarials, antipyretics, painkillers, and broad-spectrum antibiotics) was frequent and resulted already by mid-March 2019 in stock ruptures of antimalarials, antipyretics, and painkillers in hospital and private pharmacies. The affected patients complained of high out-of-pocket costs ranging around 45 USD (while 73% of the DRC population lived on less than 1.90 USD a day—World Bank 2018) for the treatment of an acute episode.

## 4. Discussion

Our field investigations confirmed the spread of the CHIKV outbreak that started around November 2018 in Kinshasa province [19] to the western end of Kongo Central province, in the densely populated portal city of Matadi. Overall, our results are indicative of a large outbreak and no/or limited prior exposure to chikungunya virus in the investigated areas. The total caseload remained unknown due to the lack of systematic surveillance. In addition, for the Matadi health zone, the reported number of clinical suspects (N = 2686) does not represent the true extent of the outbreak because of the population being reluctant to seek medical care in formal health structures due to costs, limited diagnostic resources, absence of data collection in private clinics, and the range of CHIKV illness severity. Exchanges with local health authorities, interviews with patients and caregivers, and informal discussions in the field confirmed these findings and provided insight into general perceptions, misconceptions, and knowledge gaps about the transmission and nature of CHIKV disease, as well as how health workers and communities were coping with it.

In terms of diagnosis, the yield of CHIKV PCR was logically highest in those presenting early after the onset of clinical symptoms (yield of 83% if patient presented within 1 to 3 days post onset of symptoms). From day 5 of symptoms, CHIKV IgM had a higher diagnostic yield. This turning point is in the expected range (day 5 to 7) [11,12], though somewhat to the early end, and may indicate that, in our setting, patients were underestimating their duration of symptoms or were more tolerant to initial symptoms. In similar settings, PCR or IgM testing could thus best be applied sequentially, as generally recommended in most guidelines [31,32], starting with PCR for all those for whom symptoms started less than 5 days ago, and shifting to IgM as first test for those with symptom onset from 5 days or more. The CHIKV/malaria coinfection rates were similar to findings in other CHIKV outbreaks in malaria-endemic areas in the Republic of Congo and Sudan [33,34].

Referring samples for centralized CHIKV testing is useful for the confirmation and surveillance of outbreaks and may have clinical value for individual patients to clarify residual and chronic CHIKV symptoms after the acute episode; however, it has little value for immediate patient care because of the long delay between sampling and return of results. Furthermore, during an outbreak, once the causal pathogen is confirmed, diagnostic confirmation of non-severe clinical suspects is generally not considered necessary. However, as our data highlight, in a malaria-endemic area unfamiliar with CHIKV, health workers may not feel confident in differentiating CHIKV from other conditions when they only have malaria RDTs available in addition to their clinical assessment. In Matadi, health workers resorted very often to simultaneous therapeutic coverage of CHIKV, malaria, and bacterial infections in patients with high fever and severe asthenia. This resulted in significant costs to affected families, and the inappropriate antimicrobial prescriptions may potentially contribute to antimicrobial drug resistance. Therefore, it would be of value to develop and evaluate the performance of CHIKV antigen and antibody RDTs. These could then be used at the point-of-care in diagnostic and treatment algorithms of CHIKV clinical suspects in malaria-endemic areas (including adapted clinical case definitions, presence of signs of severe/critical illness, and malaria RDT). Together with community health education, these algorithms could provide the necessary support to health workers at the primary care level for differential diagnosis and to reduce overtreatment. A few RDTs have been developed, but none seems to be sufficiently characterized in terms of diagnostic performance, as of yet, to be ready for field use [35,36,37]. The more rapid succession of outbreaks in DRC, as well as in other malaria-endemic countries [38,39,40,41], and recently published evidence of possible CHIKV and other arboviruses infections in non-human primates in the area [42] highlight this need.

The entomology results showed a high infestation of *Aedes* in the affected area, though large differences were observed between the foci. More than 95% of all adults and larvae were *Ae. Albopictus*, suggesting that this species was the main driver of the CHIKV outbreak in Matadi. This is further corroborated by the presence of E1-A226V in the virus circulating in Matadi, a mutation in the envelope gene that facilitates the spread of CHIKV in this vector [19]. These results are in line with our findings in Kinshasa and Kasangulu and a concurrent CHIKV outbreak in two major cities of the Republic of Congo (Brazzaville and Pointe-Noire) where the CHIKV outbreak was also linked to a massive colonization by *Ae. albopictus* and the viral E1-A226V mutation [19,43]. Overall, these data suggest a scenario of vector-switching where CHIKV is adapting to the increased presence of the invasive *Ae. albopictus* in the region. In addition, our result adds to the rare evidence of vertical CHIKV transmission in the vector population during outbreaks [1]. Although there have been occasional reports from surveillance and laboratory confirmations of vertical CHIKV transmission in a vector [44], this transmission route is rather seen as an exceptional phenomenon with no significance in natural transmission cycles.

Our work has several limitations. The characterization of the outbreak remained partial due to the lack of complete outbreak surveillance data. The investigation team was not mandated to introduce line-lists for clinical suspects, and also the provincial MoH was limited in its action as the outbreak was not officially declared. Our study population was probably biased toward less severe cases as we only included ambulatory patients. The level of detail of the clinical evaluation was limited by the conditions of a mobile clinic, and opportunities were missed to prospectively follow up this group of patients to document the chronic burden (socio-economically and clinically) of a CHIKV outbreak in this community. A recent systematic review and meta-analysis suggests that chronicity might be less frequent with the East Central South African (ECSA) CHIKV genotype, which is the strain that caused this outbreak [5,19]. There were, however, very little data available from ECSA CHIKV cohorts for this review, and anecdotal reports from Matadi pointed to a significant prolonged burden, which warrants further investigation in future outbreaks. It would also have been interesting to explore, in the interviews, how receptive the community would have been toward vector control initiatives.

The transmission and control of infectious diseases is a complex matter, affected by multiple biological, environmental, behavioral, and social factors. Understanding how all these factors interact requires the involvement of researchers and stakeholders across multiple disciplines. Our outbreak investigation approach was exemplary on this and succeeded because of this multidisciplinary approach to collect a lot of valuable data in a short period of time.

## 5. Conclusions

Our investigation confirmed a first major CHIKV outbreak in Matadi, with *Ae. albopictus* as main driver. The health sector was not well prepared for the health education, surveillance, and treatment needs of such an explosive outbreak. Given the increasing frequency of outbreaks and the expansion of the vector, consideration should be given to investing in surveillance systems for arboviral diseases and the vector at national level or in sentinel sites, the development of point-of-care diagnostics and appropriate clinical care, and vector control strategies.

## Figures and Tables

**Table 1:** Socio-demographic characteristics and malaria results of the clinical suspects seen at the mobile clinics and outpatient department during the investigation, by CHIKV infection status.

	Missing Values	Total N = 220	Acute CHIKV N = 183	No Acute CHIKV N = 37	*p*-Value
**Gender**	0				0.70
Male		84 (38.2)	69 (37.7)	15 (40.5)	
Female		136 (61.8)	114 (62,3)	22 (59.5)	
**Age** (years): median (IQR)	7	23 (10–38)	23 (11–39)	20 (6–33)	0.05
**Age groups**	7				0.02
<5 years		18 (8.5)	10 (5.7)	8 (21.6)	
5–10 years		27 (12.7)	21 (11.9)	6 (16.2)	
10–15 years		32 (15.0)	30 (17.1)	2 (5.4)	
15–55 years		126 (59.2)	106 (60.2)	20 (54.1)	
≥55 years		10 (4.7)	9 (5.1)	1 (2.7)	
**Days of symptoms**: median (IQR)	30	3 (2–5)	3 (2–5)	3 (2.5–4)	0.96
**Sites**	0				0.52
Mobile clinic Soyo Safari, Matadi HZ		82 (37.3)	66 (36)	16 (43)	
Mobile clinic Camp Molayi, Nzanza HZ		80 (36.4)	66 (36)	14 (38)	
Outpatient consultations		58 (26.3)	51 (28)	7 (19)	
**Malaria**	0				0.26
Positive Malaria RDT		44 (20)	34 (18.6)	10 (27.0)	

CHIKV = chikungunya virus. HZ = health zone. RDT = rapid diagnostic test. Acute CHIKV: clinical suspect with positive CHIKV-PCR and/or positive CHIKV IgM.

**Table 2:** Entomological data of the outbreak investigation in Matadi per neighborhood in which *Aedes* mosquitos were captured.

Location	Stage	*Ae. albopictus (n)*	*Ae. aegypti (n)*	Container Index (%) ^1^	House Index (%) ^2^	Breteau Index ^3^
Soyo Safari	Adult	41	0			
	Larvae	307	0	19	49	85
Kinkanda	Adult	410	0			
	Larvae	91	7	11	31	32
Camp Molayi	Adult	44	4			
	Larvae	346	23	9	5	5

*n* = number of Aedes captured as or reared up to adults. ^1^ Number of containers positive for immature stages of *Aedes* spp. per 100 inspected containers. ^2^ Number of houses positive for at least one container with immature stages of *Aedes* spp. per 100 inspected houses. ^3^ Number of containers positive for immature stages of *Aedes* spp. per 100 inspected houses.

**Table 3:** Estimation of the chikungunya infection rates of *Aedes albopictus* in the study neighborhoods.

Place	Stage	CHIKV Positive/Total Pools	ML Estimated Minimum Infection Rate (95%CI)	Number of Mosquitoes per Pool ^2^	Ct-Values of Positive Pool(s) ^3^
Soyo Safari	Adult ^1^	1/1	not possible	**(41)**	(19)
	Larva	2/6	0.82 % [0.15–2.93]	(**50,50**,20,50,50,50)	(43,37)
Kinkanda	Adult male	2/4	1.31 % [0.25–5.26]	(**50,50**,50,30)	(37,20)
	Adult female	2/5	0.93 % [0.18–3.27]	(50,50,50,**50,30**)	(29,17)
	Larva	0/2	0.00 % [0.00–2.38]	(53,35)	
Camp Molayi	Adult male	0/0	-	-	-
	Adult female	1/1	not possible	(**44**)	(17)
	Larva	0/7	0.00 % [0.00–0.88]	(50,50,50,50,50,50,46)	

ML = Maximum likelihood. ^1^ Adults in Soyo were not differentiated by sex due to difficult fieldwork conditions at the start; ^2^ Pools in bold and underlined indicate CHIKV-positive pools. Pools were only considered to be CHIKV positive if they were positive after two independent extractions and PCR runs. ^3^ The values represent averages of the obtained Ct-values.

## Data Availability

De-identified data are available after personal email request to the authors Anja De Weggheleire (adeweggheleire@itg.be) and Antoine Nkuba-Ndaye (antoinnkuba@gmail.com).

## Appendix A

**Interview guide for patients or their parent (in case of a child)**

Introduction

The conversations/interviews take place in the setting of a mobile clinic organized in the community in the immediate neighbourhood of the health centre. At the end of the consultation, the patient (and/or his parent in case of a child) is referred for a short interview. The interviews take place in a quiet corner at the same location and are kept short (about 20 to 30 min per person) to avoid overburdening the patients (as they are feeling ill at that moment).

The researcher introduces himself to the patient, asks him/her how it goes, and explains briefly why and about what he would like to ask some questions and how long this will take. He ends by asking whether the patient is willing to participate in the interview.

Guiding questions for the interview

(Questions are meant as prompts, to be used if the topics do not spontaneously arise during the conversation. They will not necessarily be asked in any specific order.)

**Topic 1: Current episode of illness (fever/arthralgia, etc) → to start the conversation**

- What type of signs/symptoms do you experience? Since when? Depending on the duration of symptoms, explore what the patient already did/not did in terms of seeking ‘diagnosis and care’ (formal/informal sector)?
- Did you ever experience the same complaints before? What did you think it was before coming here to see the doctor?
- Are there any other persons that you know who have the same complaints?
- What did the doctor explain? What is the diagnosis according to the doctor? What are your thoughts on this? Had you heard before already talking about chikungunya?

**Topic 2: Origin/cause of the outbreak**

- Where do you think this disease comes from? What do they say in the community?
- Provide explanation that chikungunya virus is transmitted by mosquitoes that bite during the day, so different ones than the mosquitoes transmitting malaria. Explore whether the patient finds this a plausible explanation, and if not, why not.

**Topic 3: Prevention and clinical management of chikungunya virus disease**

- This phenomenon of acute fever with strong pain in the joints is now circulating since a few weeks in the town of Matadi. Did you try to protect yourself from it? If yes, how did you do that?
- Did others in your community do other things to prevent the disease? Can you explain?
- If not yet mentioned spontaneously, ask about the methods mentioned by the health care workers (as the bracelets made from thin twigs).
- What did the other people in your community who had similar symptoms do? Where did they get treatment? Which type of treatment?

Rounding Up

- The researcher asks whether the patient has any questions he/she would like to ask, or if he would like to come back on anything he/she said previously during the conversation, or if he/she has anything else to add.
- Thank the participant.
